# CD36 Inhibitors Reduce Postprandial Hypertriglyceridemia and Protect against Diabetic Dyslipidemia and Atherosclerosis

**DOI:** 10.1371/journal.pone.0037633

**Published:** 2012-05-25

**Authors:** Alain Geloen, Lionel Helin, Benjamine Geeraert, Eric Malaud, Paul Holvoet, Gerard Marguerie

**Affiliations:** 1 INSERM U1060, CarMeN laboratory, Lyon University, INSA-Lyon, IMBL Villeurbanne, France; 2 Atherosclerosis and Metabolism Unit, Department of Cardiovascular Sciences, KU Leuven, Leuven, Belgium; 3 Arteria, Caissargues, France; Univ of Bradford, United Kingdom

## Abstract

CD36 is recognized as a lipid and fatty acid receptor and plays an important role in the metabolic syndrome and associated cardiac events. The pleiotropic activity and the multiple molecular associations of this scavenger receptor with membrane associated molecules in different cells and tissues have however questioned its potential as a therapeutic target. The present study shows that it is possible to identify low molecular weight chemicals that can block the CD36 binding and uptake functions. These inhibitors were able to reduce arterial lipid deposition, fatty acid intestinal transit, plasma concentration of triglycerides and glucose, to improve insulin sensitivity, glucose tolerance and to reduce the plasma concentration of HbAc1 in different and independent rodent models. Correlation between the anti-CD36 activity of these inhibitors and the known pathophysiological activity of this scavenger receptor in the development of atherosclerosis and diabetes were observed at pharmacological doses. Thus, CD36 might represent an attractive therapeutic target.

## Introduction

CD36 is a member of the scavenger receptor family with a broad cell type expression. The specificity of this receptor for oxidized lipoproteins (ox-LDL) is extensively documented [1]–[4]. This receptor is up regulated by ox-LDL in macrophages and contributes to the formation and accumulation of foam cells at sites of arterial lesions during early and late atherosclerosis. This concept was validated by the finding that mice with double CD36 and ApoE deficiency exhibited a greater than 77% decrease in aorta lesions and 50% decrease in aortic sinus lesions despite the induction of a very high atherogenic milieu [5]. This phenomenon was explained by the fact that recruitment and accumulation of foam cells at sites of lesions were considerably reduced in animals lacking CD36 [6], [7]. Such a conclusion was however challenged by the observation that combined deficiencies in scavenger A and CD36 functions did not ameliorate atherosclerosis in hyperlipidemic mice [8].

The role of CD36 in the binding and transport of long chain fatty acid (LCFA) in enterocytes and adipocytes is also well documented [9]–[12]. The protein is involved in the control of the intestinal transit of cholesterol, triglycerides (TG) and fatty acids (FA) [13]–[15]. CD36 deficiency can also rescue lipotoxic cardiomyopathy [16] and control hepatic triglycerides storage and secretion [17]. Lipid binding to CD36, at the early stage of intestinal lipid absorption, stimulates and controls chylomicron secretion [14], [15]. Thus, CD36 has a broad implication in FA membrane transport and may possibly be involved in the metabolic aspects of dyslipidaemia [17]. Observation that CD36 may regulate downstream signalling in enterocytes and stimulate chylomicron synthesis supports this hypothesis [18]. This concept is however questioned by the consistent observation that CD36 gene deletion does not affect plasma TG concentration, LCFA uptake and TG re-esterification in mouse proximal intestine and that postprandial plasma TG concentration is increased in CD36 deficient humans [18], [19]. Therefore, the direct role of CD36 in the intestinal absorption of FA and its pathological hyperlipemia consequence remains an open question.

In addition to its potential implication in atherosclerosis and dyslipidaemia, independent studies have suggested that CD36 may also be directly or indirectly involved in diabetes [20], [21]. CD36 deficient humans were reported to have insulin resistance [19], [22]. CD36 gene knock out, however, did not induce insulin resistance in mice [5]. Rather, insulin sensitivity was increased in CD36^−/−^ skeletal muscle [23]. Furthermore, defective insulin signalling was shown to be associated with increased CD36 expression in macrophages [24]. In addition, ox-LDL produced a dramatic reduction of Glyceraldehyde-3-phosphate deshydrogenase in smooth muscle cells resulting in a marked reduction of glucose usage [25]. Together, these observations suggest that CD36 is inversely correlated with insulin sensitivity and plasma lipoproteins. In contrast, animals over expressing CD36 in muscle exhibited decreased plasma concentrations of triglycerides and increased plasma insulin and glucose concentrations [26] and CD36 deficiency induced insulin resistance in the liver of these animals [23]. Therefore, opinions concerning a direct or indirect role of CD36 in insulin resistance and the development of type II diabetes are diverging.

In summary, the preponderance of evidence suggests that CD36 is a central receptor for the detection, accumulation and metabolism of lipids and fatty acids in different cells and tissues. CD36 could then function as a molecular bridge between the development of dyslipidaemia and insulin resistance [21]. If so, it may represent an interesting therapeutic target for the treatment of atherosclerosis, type II diabetes and obesity and their associated cardiovascular diseases. In support with that hypothesis, we show that small molecules with anti-CD36 activity can reduce postprandial hyperlipidaemia and protect against type II diabetes and atherosclerosis.

## Materials and Methods

### Cell Culture

HEK 293 cells (ATCC) were transfected with a full length human CD36 cDNA to obtain a permanent CD36 expressing cell line. Expression of a functional CD36 at the surface of the cells was characterized by flow cytometry using monoclonal anti-CD36 antibody (FA6.152, Abcam). Human THP-1 cells (ECCAC) were cultured in RPMI-1640 medium supplemented with 10% fetal bovine serum (FBS), 200 mmol/L L-Glutamine, 100 Unit/mL penicillin and 100 µg/mL streptomycin. HEK 293 wt and HEK293-CD36 cells were cultured in Dulbecco’s modified Eagle’s medium (DMEM) supplemented with 10% FBS, 1 mmol/L Sodium Pyruvate, 0.1 mmol/L non essential amino acids, 200 mmol/L L-Glutamine, 100 Unit/mL penicillin and 100 µg/mL streptomycin. CD36 expressing cells were all time cultured in presence of 300 µg/mL selection antibiotic G418 (sigma). For THP1 experiments, cells were differentiated into macrophages in RPMI-1640 medium supplemented with 5% FBS and 10^−7^ mol/L of PMA for 24 hours at 37°C, 5% CO_2_. For experiments with HEK293 and CD36 expressing HEK293, cells were plated in 96-black plate coated with poly-D-lysine for 48 hours at 37°C, 5% CO_2_ in DMEM containing 10% FBS.

**Figure 1:**
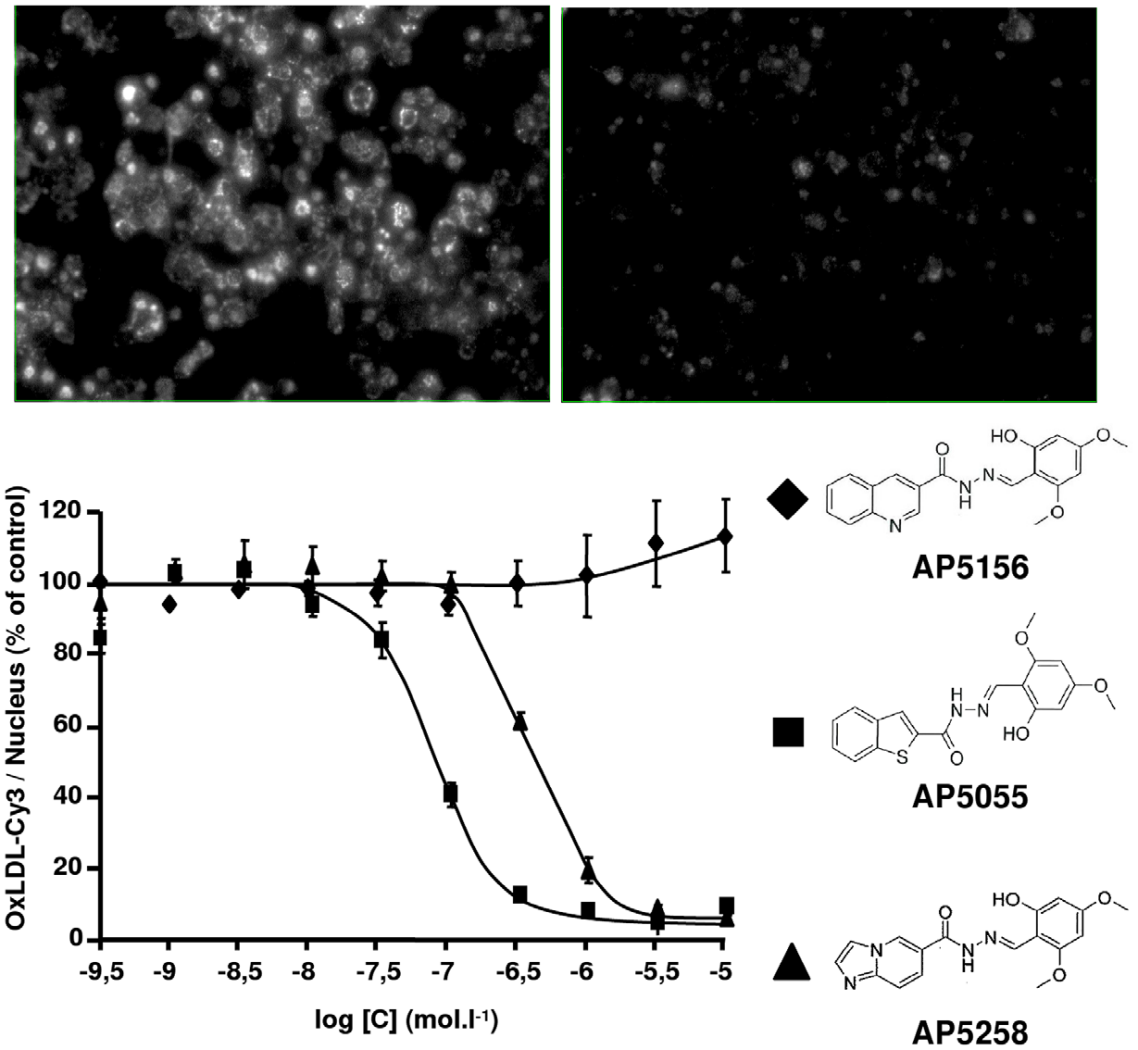
Chemical structures and activities of the CD36 inhibitors AP 5055, AP 5258 and the negative analog AP5156. Dose dependent inhibition of ox-LDL uptake and accumulation by THP1 cells at 37°C. Uptake was measured as the cyanin3 labeled ox-LDL uptake at constant cell number.

### Lipoproteins Preparation

LDL were prepared by discontinuous density gradient ultracentrifugation from normal human plasma provided by Etablissement Français du Sang. LDL were oxidized with 5 µmol/L CuSO_4_ for 15 hours at 37°C. Oxidation was stopped by addition of 40 µmol/L butylated hydroxitoluene (BHT) and 100 µmol/L diethylene triamine penta acetic acid (DTPA), then extensively dialyzed against phosphate buffered saline containing 100 µmol/L DTPA. Oxidized-LDL were labeled with cyanine3 Mono NHS ester. Oxidation was controlled by electrophoretic mobility and the measurement of optical density at 234 nm corresponding to the formation of conjugated dienes.

### LCFA Preparation and Endocytosis Experiments

Fat-free BSA, Bodipy fl C16 (Molecular probes), palmitate and Phloretin were purchased from commercial sources. Both the albumin and palmitate concentration were 173 µmol/L. 23 µmol/L of the palmitate component were bodipy fl C16 and 150 µmol/L were non-fluorescent palmitate. Briefly, HEK293 and HEK293/CD36 cells were preincubated for 30 minutes at 37°C in serum free DMEM containing 0.1% dimethylsulfoxide (DMSO) or molecules. Following preincubation, the culture medium were replaced with phosphate buffer saline containing BSA/palmitate/bodipy fl C16 complex in presence of 0.1% DMSO or molecules. The uptake was stopped by removal of the solution followed by addition of 100 µL of an ice-cold stop solution containing 0.5% of free fatty acid albumin and 0.2 mmol/L of Phloretin. The stop solution was discharged after 2 minutes and the culture dishes were washed by deeping them three times in ice-cold incubation buffer.

The cells were fixed in 4% paraformaldehyde during 30 minutes at 4°C and nucleus were stained by incubation in buffer saline containing 1 µg/mL of Hoechst 33342 during 20 minutes at room temperature. Then, cells were washed twice in phosphate buffer saline. The Leica DMIRB microscope system was used for staining visualization. 16 images by well of cells staining with Bodipy fl C16 and Hoechst 33342 were automatically captured using a fluorescence microscope controlled by MetaMorph Software (Universal Imaging) and coupled with a CCD camera. After images analysis, results were expressed as the sum of bodipy intensity per cells number.

**Figure 2:**
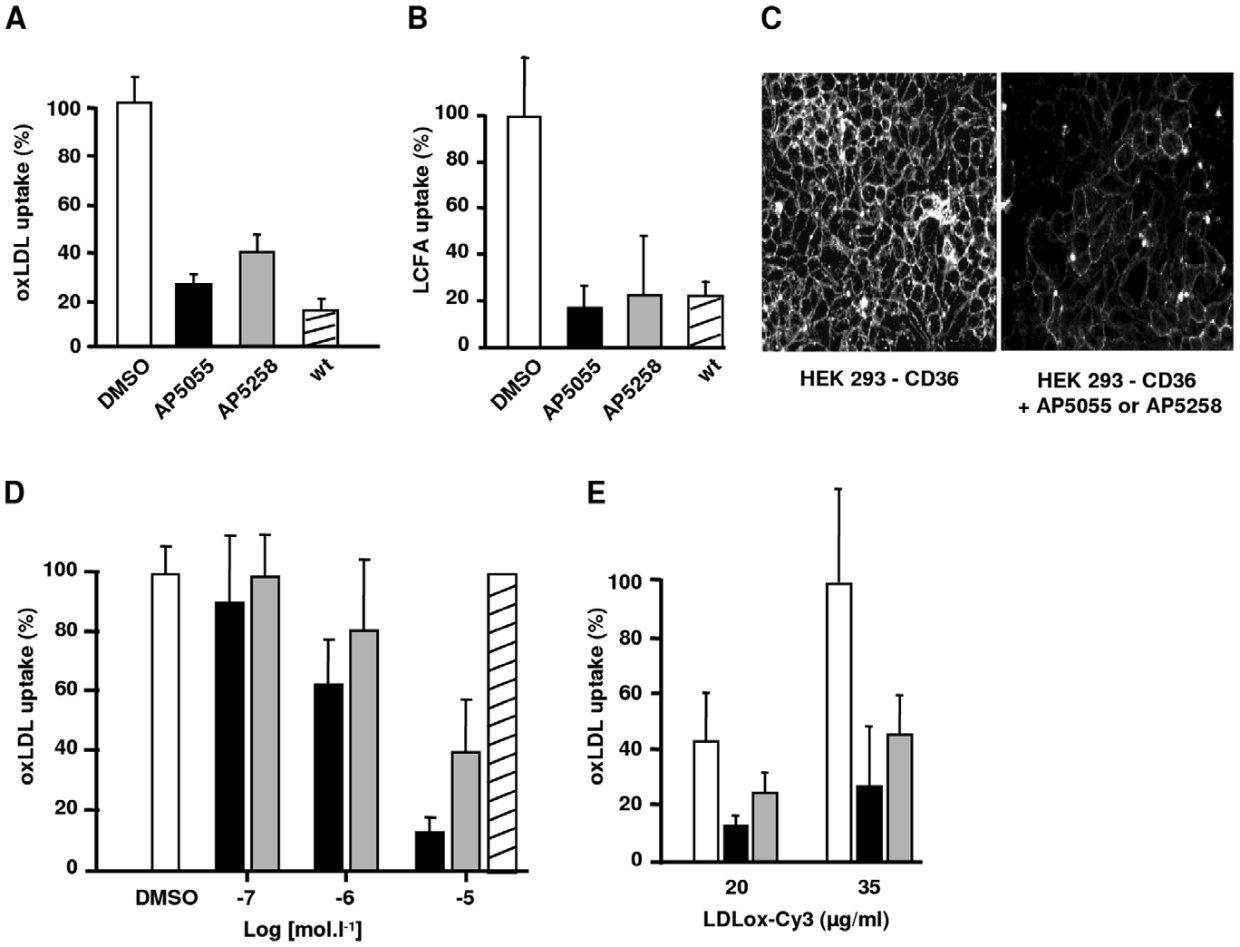
Anti-CD36 activity of AP5055 (dark) and AP5258 (grey) on CD36-HEK cells. Non transfected cells (wt), were used as control: A: ox-LDL uptake at 37°C, B: Palmitate uptake at 37°C, C: typical inhibition of ox-LDL binding at 4°C, D: dose dependent inhibition of AP 5055 and AP5258 on ox-LDL binding, AP5156 used as negative control had no effect (hatched bar), E: Comparative inhibition of ox-LDL binding by AP5055 and AP5258 at different ox-LDL concentrations.

### Ox-LDL Binding and Endocytosis Experiments

All binding experiments were performed at 4°C. HEK wt and HEK-CD36 at 80% of confluence in 96-well plates were incubated with the appropriate medium containing 1 µg/mL of Hoechst 33342 for 20 minutes at 4°C. After washing twice, cells were incubated in medium supplemented with 0.5% FBS and cyanine 3-oxidized-LDL (20 or 35 µg/mL), in the absence or the presence of increasing concentration of the compound for 4 hours at 4°C. Cells were fixed with 4% paraformaldehyde for 15 minutes at 4°C and then washed with phosphate buffered saline before fluorescence microscopy analysis.

For endocytosis experiments, the cells, at 80% confluence, in 96 well plates, were incubated with the appropriate concentration of the compound for required time at 37°C. Cells were fixed with 4% paraformaldehyde for 20 minutes at room temperature. After washing, the nucleus was labeled with 1 µg/mL of Hoechst 33342 for 20 minutes at room temperature.

Each stimulation condition for binding and endocytosis was performed at 0.1% DMSO in quadruplicate. 16 images by well of cells staining with Cyanine-3 and Hoechst 33342 were automatically captured using a fluorescence microscope controlled by MetaMorph Software (Universal Imaging) and coupled with a CCD camera. After images analysis, results were expressed as the sum of cyanine-3 intensity per cell number.

**Figure 3:**
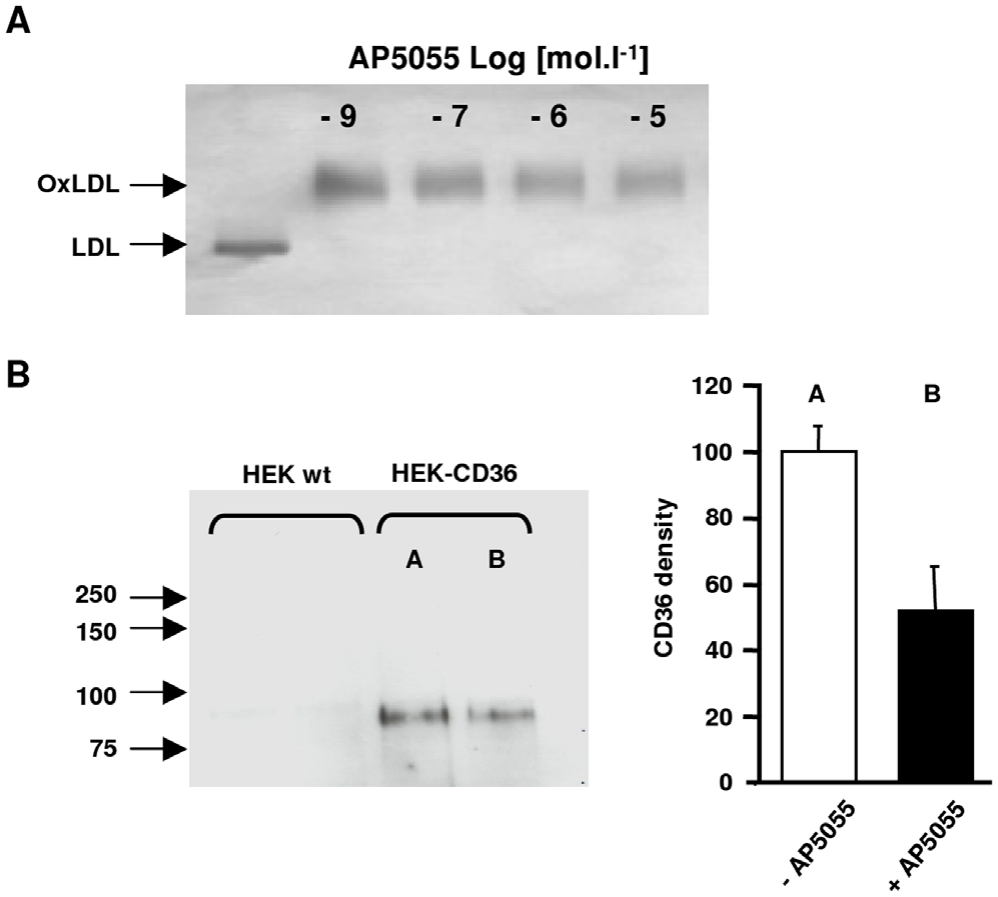
Anti-CD36 activity. Effect of AP5055 on the molecular interaction between CD36 and ox-LDL using CD36-HEK and wild type (wt) cells at 4°C. A: effect on the electrophoretic mobility of ox-LDL, B: Affinity crosslinking of ox-LDL to membrane expressed CD36, biotinylated ox-LDL were cross linked at 4°C, the ox-LDL complex was immunoprecipitated with an anti ox-LDL antibody and complex-associated CD36 was detected on immunoblot, using an anti CD36 antibody.

### Animals and Experimental Protocols

All experiments were carried out according to the guidelines laid down by the French Ministère de l’Agriculture and E.U. Council Directive for the Care and Use of Laboratory Animals (N° 02889). Experimental procedures in animals were performed in accordance with protocols approved by the Institutional Animal Care and Research Advisory Committee of KU Leuven (LA1210544) and Comité d’Expérimentation Animale of University Claude Bernard, Lyon1 (BH 2012–23).

### Rodent Models

#### Atherosclerosis mouse model

Homozygous LDL-receptor deficient (LDL-R^−/−^) mice, heterozygous leptin deficient mice (ob/+) and C57BL/6 mice were purchased from Jackson Laboratories. Double knockout mice (DKO) characterized by both leptin deficiency (ob/ob) and LDL-R deficiency were obtained by crossing as previously described [26]. The phenotypic attributes of these DKO mice have been previously characterized in details [26]. All mice were maintained in controlled conditions (22°C with a fixed 12/12 hour light/dark cycle) and fed standard chow containing 4% fat (Pavan Service). Mice were daily treated for 12 weeks with intraperitoneal (IP) injection of AP5055 at 1 mg/kg supplemented with 1% methyl cellulose or vehicle (10% DMSO).

**Figure 4:**
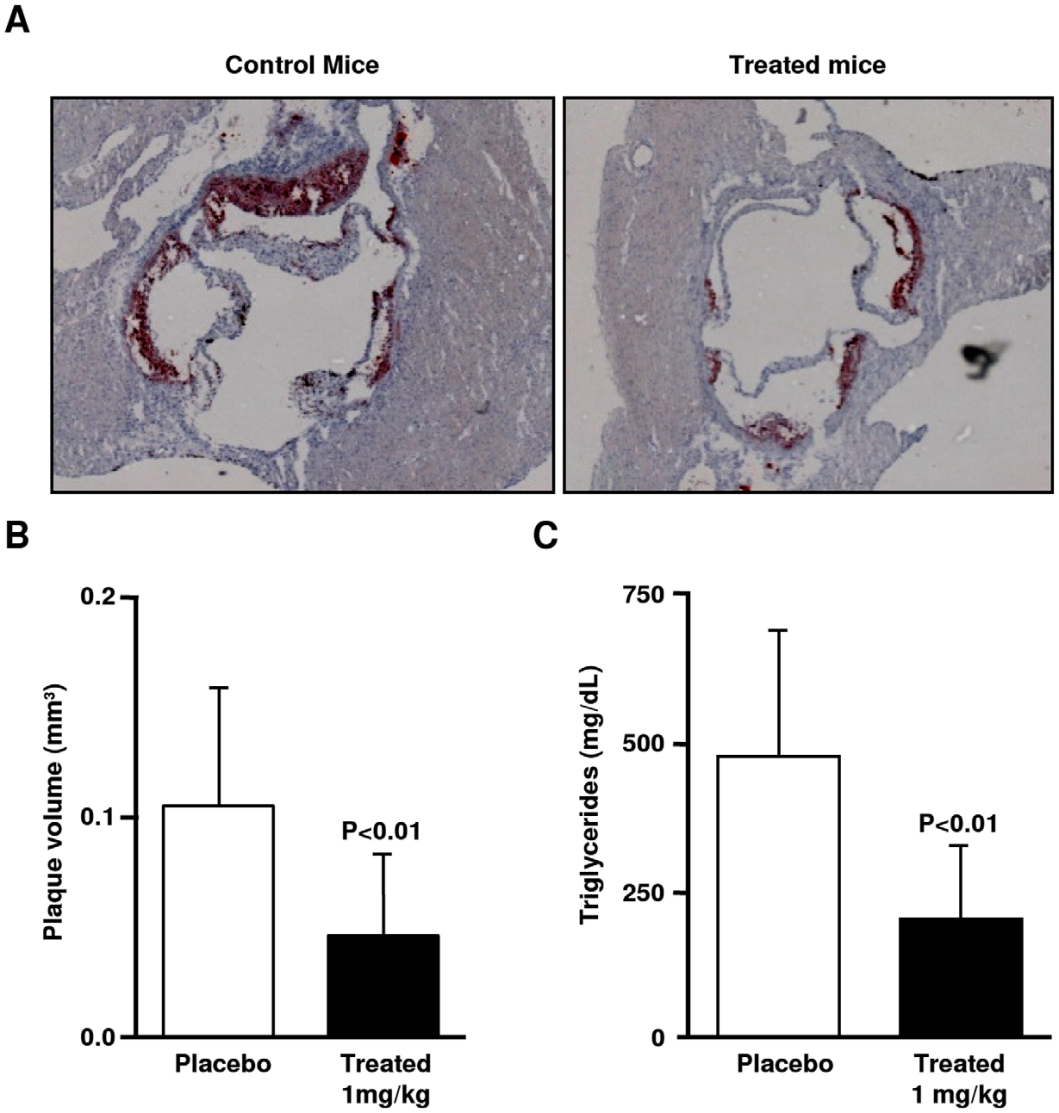
Protection against atherosclerosis. Effect of AP 5055 on the development of atherosclerosis in double LDL-R and Leptin deficient mice (DKO). A: lipid deposition in the aorta, B: plaque volume, C: plasma TG concentration.

**Figure 5:**
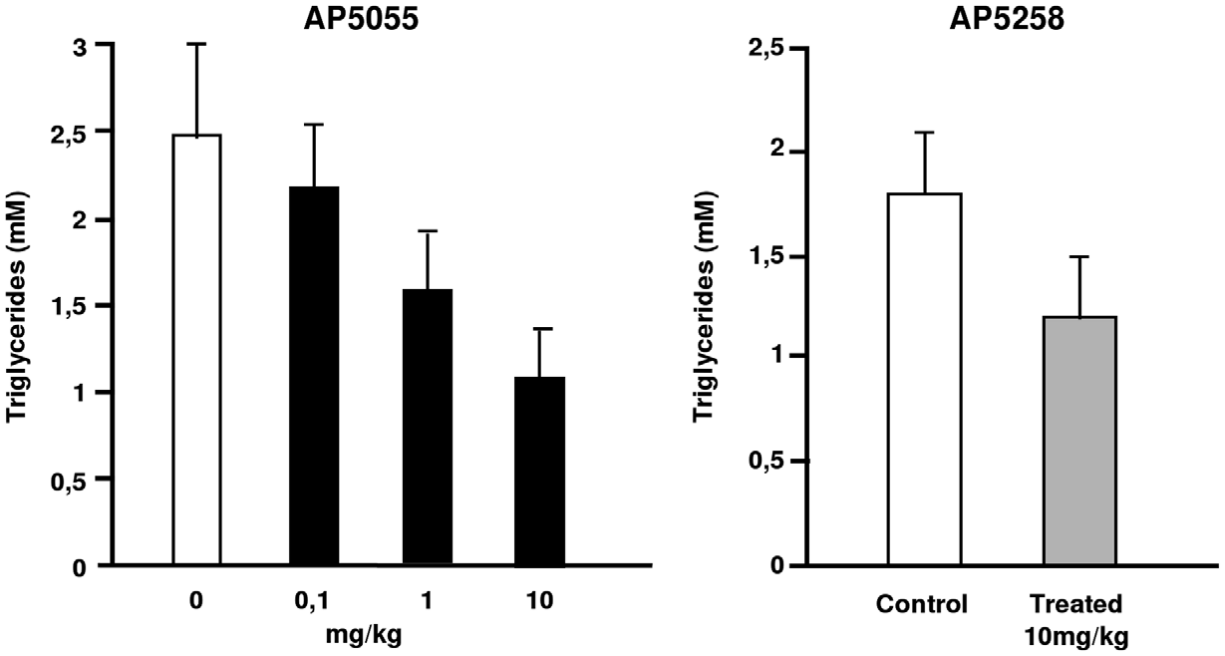
Reduction of plasma triglycerides. Comparative effect of CD36 inhibitors on the plasma concentrations of TG in different rat models, A: Dose dependent reduction in a fructose fed rat, AP5055 was administrated at different doses for 3 w (n = 12), B: AP5258 was administrated to diabetic ZDF rats (C = Control, T = Treated) for a period of 2w at 10 mg/kg (n = 8).

#### Diabetic rat model

Male Wistar Han rats (250–260 g, Elevage Janvier, Le Genest St Isle, France) were used. The fructose diet-inducing diabetic model was previously well described. Briefly, adult rats were fed with a standard chow (A04, UAR, Villemoisson, France) and given *ad libidum* 10% fructose solution for 21 days. After a 3-week diet, diabetic rats were daily treated by IP administration of different doses of the compound, mixed with 2% DMSO or PEG 300 for 21 days. Plasma samples were obtained in the fed state *via* the tail and prepared for concentration measurements of TG levels.

#### Prediabetic ZDF rat model

Male Zucker diabetic fatty rats (ZDF-Lepr fa/Crl) (5 weeks old) (Charles River, France) were fed maintenance rodent diet 2016 C (Harlan France). These rats become diabetic between 7 and 10 weeks. After an adaptation period of 2 weeks, vehicle, being either PEG 300, or CD36 inhibitor (50 mg/kg) were administrated, during the rise of plasma glucose. Blood samples were obtained from tail vein and plasma glucose and HbA1c were determined throughout the 3 week treatment. Glycated hemoglobin was measured using the direct Enzymatic HbA1c Assay™ (Diazime laboratories, CA, US). A second experiment was carried out on older Zucker rats (18 weeks), in which rats received IP injections of AP5258. Oral glucose tolerance and insulin tolerance test were performed at the end of the treatment period. Blood samples were obtained from tail vein and concentrations of plasma glucose and triglycerides were determined during the time course of the treatment.

**Figure 6:**
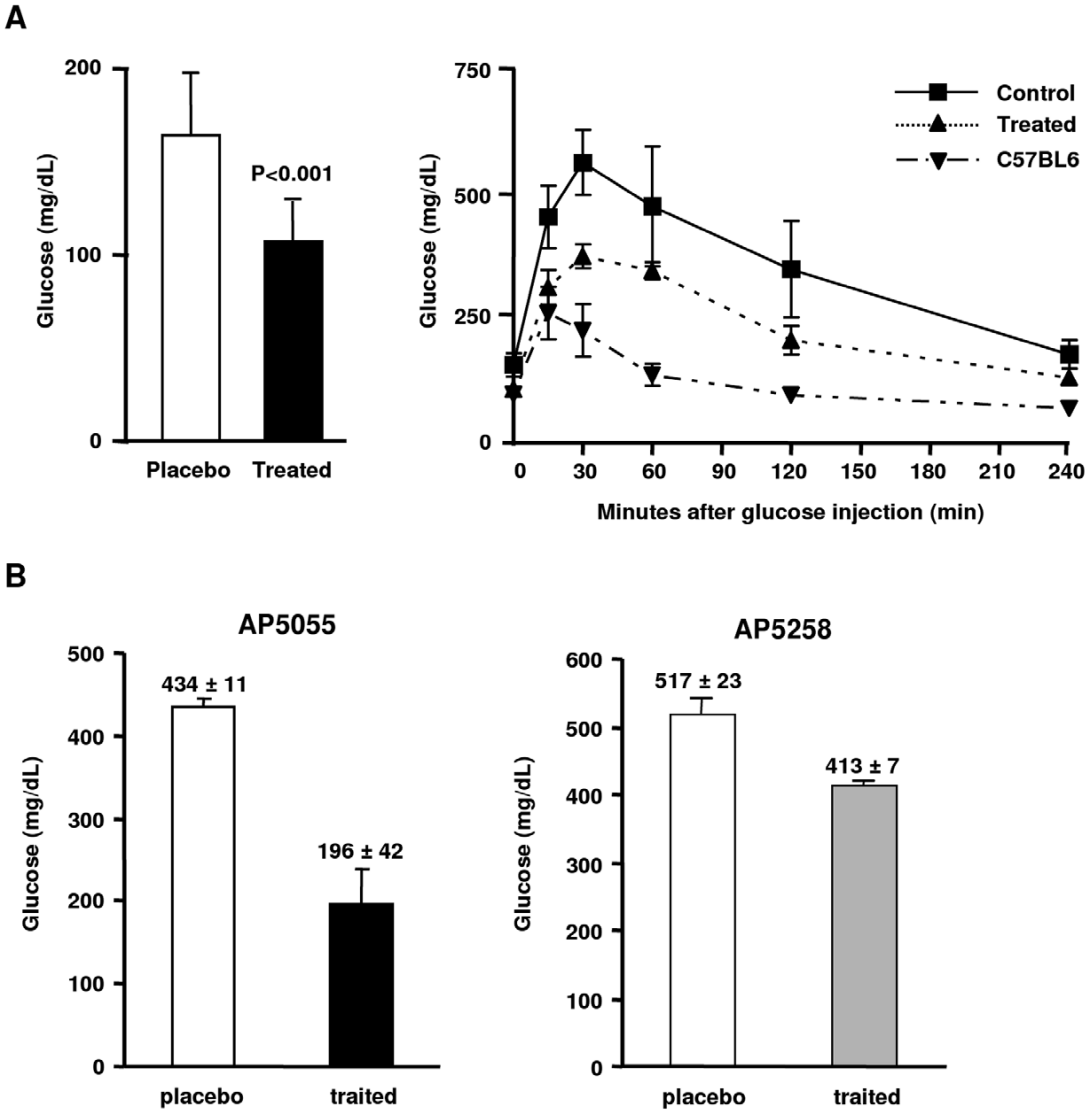
Effect of CD36 inhibitors on insulin resistance. A: Effect of AP5055 (1 mg/kg), on the glucose level and the glucose tolerance in the DKO mice, B: effect of AP5055 and AP 5258 on the plasma glucose in a ZDF rat (40 mg/kg, 3 w, n = 8).

**Figure 7:**
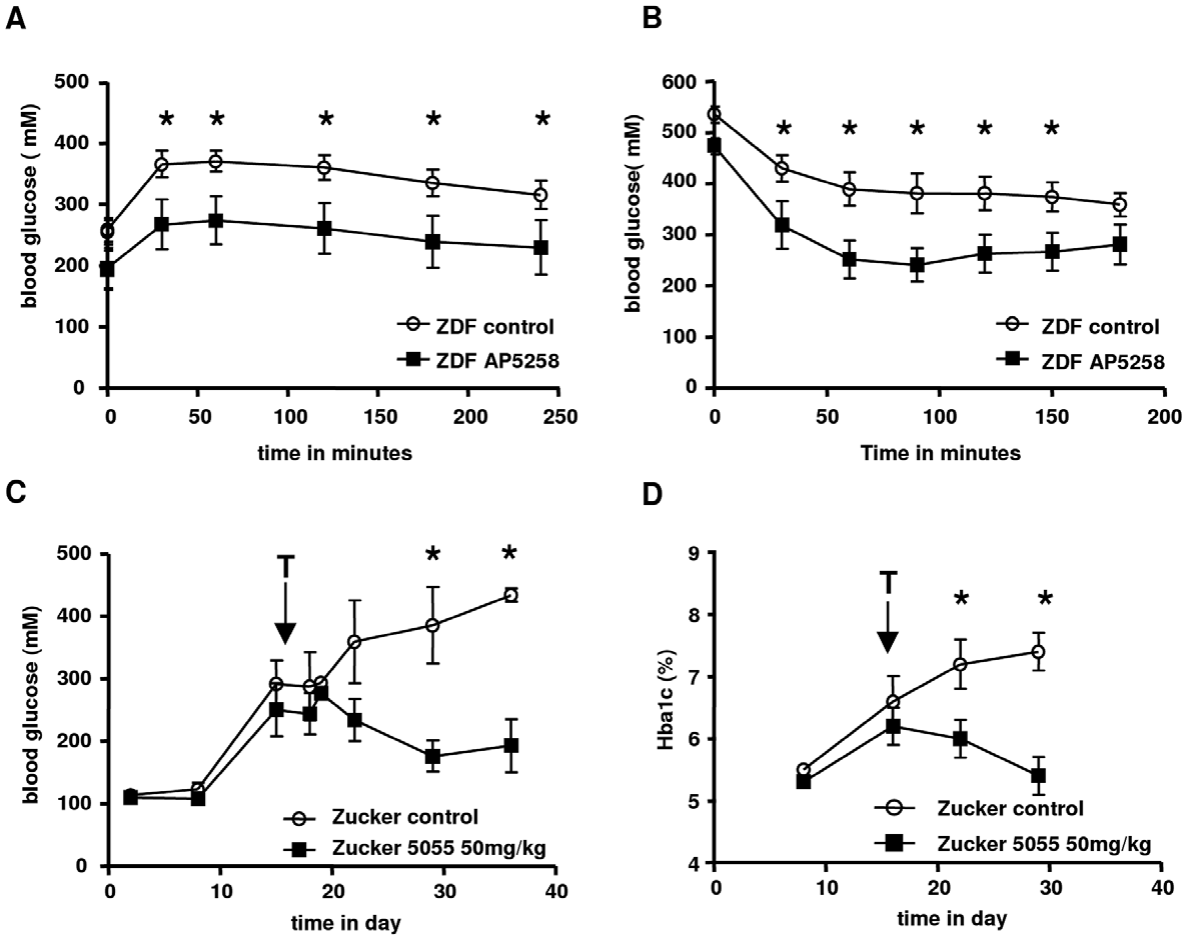
Effect of CD36 inhibitor on the metabolic syndrome parameters. A: effect of AP5258 on OGT, B: Effect of 5258 on insulin sensitivity, C: Effect of AP5055 on plasma glucose, D: Effect of 5055 on the plasma concentration of HbAc1.

**Figure 8:**
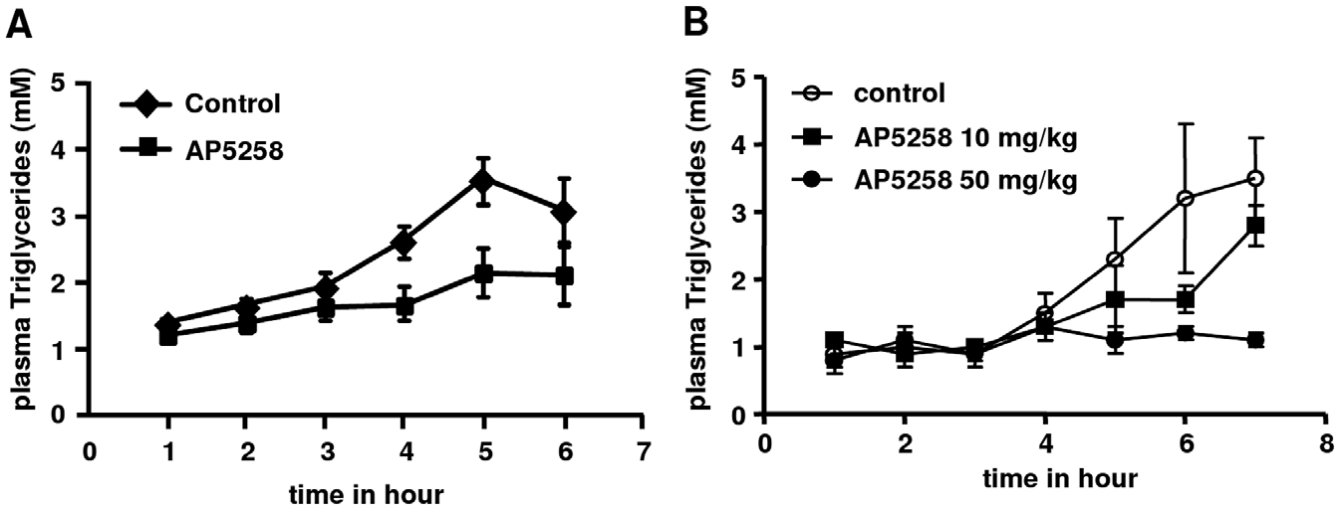
Reduction of FA intestinal transit. Oral administration of anti-CD36 inhibitor reduces post prandial HTTG following a gastric olive oil challenge. A: kinetics of the TG intestinal transit in the plasma in control animals or treated animals (AP5258, 50 mg/kg n = 12), B: Dose dependent effect on Plasma TG (n = 3).

**Table 1:** Inhibition of ppTG in the plasma at four hours following the olive oil gavage following administration of 50 mg/kg of active (AP5055, AP5258) or inactive (AP5156) analogues.

Plasma postprandial TG (mM) at 4 h	
**Vehicle**	**2.8±0.6**
**AP5156**	**2.6±0.6**
**AP5055**	**1.7±0.8**
**AP5258**	**1.7±0.6**

#### Postprandial analysis in Sprague Dawley (SD) rats

Sprague-Dawley rats (SD, 225–250 g) (Charles River, France) were fed a rodent maintenance global diet (2016C, Harlan France). Postprandial plasma TG concentrations were determined during 6 hours after an olive oil test. Briefly, the animals were fasted overnight for 16 hr and then forced fed with 1 mL of olive oil. Blood samples were collected every hour through the tail vein. Rats received either vehicle or active compound (50 mg/kg) in PEG 300. Plasma TG were measured with a commercial kit (TG PAP 150, Biomérieux, France.

### Quantification of Atherosclerotic Plaque Lesions

After 12 weeks treatment with AP5055, the extent of atherosclerosis was evaluated by serial cross sections from aortic root. Immunohistochemical and morphometric analysis were performed on 7-µm cryosections. Lipid staining with oil red O, with mAb4E6, smooth muscle cells with an α-actin specific antibody (Dako) and macrophages with an antibody against mouse Mac-3 antigen (Pharmingen) were performed. Immunostained sections were blindly analyzed with Quantimet 600 image analyzer (Leika).

### Oral Glucose Tolerance Test (OGTT)

Rats were fasted for 16 h before glucose solution (2 g/kg body weight) was administered orally. Blood was drawn from a tail vein at 10, 20, 30, 60, 90 and 120 min after the administration of glucose. Plasma glucose concentration was measured with a commercial glucometer (Accu-Chek, Roche, France).

### Statistical Analysis

All data are reported as mean ± standard error of the mean. Plasma concentration assays were performed on 8 to 12 animals per group. Statistical significance between control treated animals was determined by analysis of variance (ANOVA). The Fisher protected least significant difference, post hoc test was used for group comparisons. Statistical significance was set at p<0.05.

## Results

### Identification and Characterization of Anti CD36 Inhibitors

To identify chemical compounds with anti-CD36 function, a CD36-expressing HEK-293 cell line was established for high throughput screening of large chemical libraries. One series of pharmacophore was identified and optimized for their capacity to inhibit binding, uptake and accumulation of ox-LDL by THP1 cells. Two members of this series, named AP5055 and AP5258 produced a significant inhibition of foam cells formation with IC50 of 100 nM and 500 nM respectively and were selected for further studies. This inhibition was observed at constant nucleus number (Figure 1). One analog of the same series, AP5156, with similar chemical structure was inactive indicating the presence of a structure-function relationship within this chemical series.

HEK-CD36 cells interacted with both LCFA and oxidized lipoprotein particles, stored these particles and accumulated lipid rich vesicles in a CD36-dependent way. This cell line was further utilized to explore the anti-CD36 activity of these chemical entities. When performed at 37°C, lipid vesicles accumulation by these cells was significantly inhibited by both AP5055 and AP5258 with IC50 similar to that observed with THP1 cells (Figure 2A). Similarly, both AP5055 and AP5258 inhibited palmitate cellular transfer to a level comparable to that observed with non-transfected wild type cells (Figure 2B). Both inhibitors produced a dose dependent inhibition of CD36-dependent binding to the membrane of these cells with IC50 of 1±0.1 µM and 5±1 µM respectively (Figure 2C and 2D). The analog AP5156 used as a negative control, had no effect on this binding, up to a concentration of 10^−4^ M (Figure 2D).

The compounds AP5055 and AP5258 were then utilized to further explore the receptor inhibitor activity of this chemical series. Different experiments indicated that these inhibitors are receptor rather than oxLDL directed. First, the compounds did not affect the electrophoretic mobility of the complex at any concentration tested as illustrated in Figure 3A. Second, both AP5055 and AP5258 had no effect on the CD36-independent binding as observed with wild type HEK cells. This level of CD36-independent binding never exceeded 15% on the wt HEK cells. Third, when bound biotynilated-oxLDL was affinity cross-linked to the HEK-CD36 membrane, then immunoprecipitated with an anti-biotin monoclonal antibody, and analyzed by western blotting with an anti-CD36 monoclonal antibody, after reduction to quantify bound receptors, the compound produced a significant inhibition of the oxLDL-CD36 complex cross-linking. Figure 3B exemplifies the results obtained with AP5055. AP5258 had a similar effect. Non transfected wt HEK cells did not crosslink oxLDL. Finally, compound-induced inhibition was dependent upon the concentration of the ligand, with an increased inhibitory capacity at greater ox-LDL concentration suggesting that AP5055 and AP5258 are noncompetitive CD36 inhibitors (Figure 2 E). Altogether, these experiments demonstrated that both molecules were inhibitors of the oxLDL and LCFA receptor functions of CD36 with AP5055 being slightly more potent than AP5258.

### Protection Against Atherosclerosis

CD36 deficient mice are protected against atherosclerosis. Therefore, the *in vivo* efficacy of the compound to protect against atherosclerosis was first examined in double LDL-R and leptin deficient mice (DKO). Figure 4 illustrates the results and exemplifies the activity obtained when AP5055 was administrated to these mice. Typical oil red O-staining of the lesions in the aortic root of treated free fed mice (24 weeks old) was compared to non-treated animals. Consistent with previously published observations [26], non-treated mice developed small fatty streaks with plaque volumes at 0.084±0.034 mm^3^ (n = 19). Daily IP injection of the compound at 1 mg/kg for a period of 12 weeks produced a significant reduction of lipid deposition as illustrated by the reduction of oil red O staining. Plaque volume was reduced to 0.045±0.032 mm^3^ (n = 12) corresponding to a 46% reduction. Concomitant with the reduction of lipid deposition, a significant decrease of plasma TG was observed (Figure 4B). TG did not change in placebo treated mice while AP5055 produced a greater than 50% reduction. Thus, anti-CD36 compounds are able to protect against the growth of atherosclerotic plaque at an *in vivo* concentration compatible with the *in vitro* activity of this molecule (∼10^−5^ M). Reduction of the plasma level of TG was however unexpected because CD36-deficient mice were reported to have increased levels of plasma TG (Figure 4C).

To verify that this result was not model specific and was not due to the double knock-out of both the LDL-R and leptin genes in the DKO mouse model, the effect of anti-CD36 molecule on plasma TG concentration was examined in an independent diabetic fructose fed rat model. Results that summarize these experiments are illustrated in Figure 5. When administrated (IP) at concentration ranging from 0.1 to 10 mg/kg AP5055 was able to produce a similar dose dependent reduction of the plasma TG within weeks of treatment. When using the ZDF rat model, AP5258 produced a significant reduction of the TG plasma concentration (Figure 5). The inactive analog AP5156 had no effect (result not shown). Therefore, the decrease in plasma TG correlated with the cellular activity of the compounds and was not model or analog dependent. Differences in the potency of these molecules in the different models were however observed. This may be explained by the relative stringency of the different models in terms of metabolic syndrome, the ZDF rat being less sensitive to the treatment than the mouse or the fructose fed rat model. Alternatively, the two compounds may have different metabolism.

### Protection Against Insulin Resistance

When administrated to the DKO mice at 1 mg/kg, AP5055 produced a significant reduction of the plasma glucose and improved glucose tolerance (Figure 6A).

To verify that this effect was not model dependent, AP5055 and AP5258 were administrated to the ZDF rat model to measure changes in fasting and non fasting plasma glucose. Typical results are illustrated in the figure 6B and 6C. Both molecules were able to reduce fasting and dietary plasma glucose levels after 3 w of treatment at 40 mg/kg. Greater than 50% inhibition of plasma glucose was obtained with AP5055 (434±11 vs 196±42 mg/dL), whereas AP5258 produced a 20% inhibition at the same dose (517±23 vs 413±7 mg/dL). AP5258 produced a similar correction on the oral glucose tolerance test. In addition to the induction of a better glucose tolerance, sensitivity to insulin was also improved. Typical results obtained for AP5258 are illustrated in Figures 7A and 7B.

Therefore anti-CD36 inhibitors were able to improve glucose tolerance in rodent animals with typical metabolic syndrome. The effect on glucose tolerance was objectivized by the decrease of HbAc1 observed on Zucker rat model (Figures 7C and 7D).

### Reduction of Post Prandial Hyperlipidemia

The time course of the plasma TG response to a gastric olive oil challenge is shown in Figure 8. After overnight fasting the plasma TG concentrations were similar at time 0 in the control and treated animals at an average level of 1.16±0.35 mM. The postprandial lipemia started to increase at 4 h in the non-treated animals and reached a maximum value at 6 h. When the animals were treated with AP5258 at 50 mg/kg the postprandial increase of plasma triglycerides was inhibited. This total inhibition of the intestinal transit was dose dependent since at a dose of 10 mg/kg an intermediate effect was observed (Figure 8B).

These results were consistently reproduced in different and independent series of animals. When taken at 4 hours, on a larger number of animals (n = 8), 50 mg/kg produced a greater than 40% inhibition (Table 1).

## Discussion

In the present study, correlation between the anti-CD36 inhibitor activity of small molecular weight chemicals and the known pathophysiological activity of this scavenger receptor were established. Although different mechanisms may be involved in the oral versus IP activity of these inhibitors, both administrations were able to improve the metabolic profile of defined and independent rodent models. A significant reduction of the plasma concentration of triglycerides and a better glucose usage were observed at pharmacological doses with a concomitant reduction of the atherosclerotic and diabetic consequences of these attributes.

CD36 is a well characterized FA translocase and an oxidized LDL receptor expressed in many cell types including macrophages, adipocytes, endothelial cells and enterocytes [3], [9], [10], [14], [27], [28]–[30]. Expression of this gene is ligand-binding dependent and can either be up or down regulated. For instance, ox-LDL-CD36 interaction up regulates a PPARγ-dependent CD36 gene expression in monocytes-macrophages [31] whereas interaction with FA down regulates gene expression and protein synthesis in enterocytes [18], but can up regulate the gene in adipocytes [28]. In addition, CD36 may or may not be associated with companion molecules. The Vitronectin receptor VNR [32]–[33], caveolin-1 [34], the Intestinal alkaline phosphatase IAP [35], the CD9 tetraspanin [36], [37] and the Toll-receptor complex [38] show molecular and functional associations with CD36 at the surface of cells. Therefore, genetic expression and molecular functions of CD36 are complex and controlled by membrane and tissue specific molecular associations and different cellular specific signaling pathways. This pleiotropic effect may reasonably well question the clinical relevance and safety of CD36.

While the cellular functions of CD36 are recognized, its importance in the physiopathology is less well understood and often controversial. The role of CD36 in the formation of foam cells and the growth of atherosclerotic plaques is well documented. Yet the role of CD36 as a target to combat atherosclerosis was criticized [8]. Similarly, evidences supporting a role of CD36 in intestinal fat absorption are accumulated, but contradictory observations have also been reported concerning its direct implication in intestinal lipid trafficking and the control of postprandial hypertriglyceridemia. For instance, CD36 is expressed all through the intestinal tract and is important for the metabolism and the secretion of chylomicron into the lymph [15]. The molecule is required for efficient intestinal absorption of LCFA and VLCFA [13]–[15]. Yet, CD36 deficient mice exhibit a normal level of FA absorption [15] and gene deletion does not affect LCFA uptake and TG re-esterification in mouse jejunum [18]. Therefore the potential of CD36 as a therapeutic target is debated. In the present paper we have identified small chemical molecules which have the capacity to inhibit the FA and ox-LDL receptor function of CD36. These inhibitors were able to rescue well characterized animal models from postprandial hypertriglyceridemia and atherosclerosis with a concomitant improvement of insulin resistance and glucose tolerance.

The CD36-inhibitor activity of this new chemical series was established on the following criteria. First, the molecules were selected for their capacity to inhibit ox-LDL binding, uptake and accumulation in THP1 cells. Furthermore, using CD36-transfected HEK cells the specificity of this inhibition for CD36 was demonstrated. Active members of this series were able to completely inhibit binding and uptake to levels that were similar to the non-specific binding and uptake observed with wt cells. Second, consistent with the dual function of CD36 as a receptor for two different ligands, and the non-competitive agonist activity of these inhibitors, a similar activity on LCFA binding and uptake on both THP1 and HEK-CD36 cells was measured. These results support a receptor rather than a ligand-driven inhibition. Third, analogs of the same series with close chemical structure had no effect on these cellular functions, suggesting the existence of a structure-function relationship within the members of the series. Finally, cross-linking affinity was used to demonstrate the effect of the compounds on the molecular interaction between ox-LDL and CD36. In aggregate, these new molecules were able to inhibit the CD36 receptor function both at the cellular and the molecular levels.

The first CD36 *in vivo* activity to be examined was its implication in the development of atherosclerosis using a well characterized animal model. A DKO mouse combining LDL-R and leptin deficiencies was used. This model exhibits high blood pressure together with increased plasma TG concentration, insulin and glucose. It develops atherosclerosis and represents a good model to study the physiopathology of the metabolic syndrome [26]. The CD36-antagonists used in the present study were able to reduce the growth of atherosclerotic plaques at plasma concentrations compatible with the cellular activity of these molecules. This is in agreement with the fact that CD36 depleted mice are protected against atherosclerosis [5]. Unexpectedly, a significant reduction of the plasma TG was also observed. Increased plasma TG concentration is an important factor for the development of atherosclerosis. The DKO mouse, the ZDF rat, and the fructose rat model exhibited a significant increase of the plasma TG concentration and in these animals, the compounds were able to reduce plasma TG, indicating that this reduction was not model dependent. These observations do not agree with previously published observations showing that CD36 deletion in mice impairs lipoprotein lipase-mediated TG clearance [39] and results in increased levels of plasma triglycerides. The present study demonstrates that an anti-CD36-ox-LDL and Fatty Acid binding activity has the capacity to reduce plasma triglycerides in rodent species. This reduction was in good agreement with the observed reduction of lipoprotein deposition in the aortic tree and the plaque growth. CD36 is implicated in lipid metabolism but has not yet been implicated in lipogenesis. Therefore it is unlikely that an inhibitor of CD36-binding may directly influence TG synthesis *per se*. While a pleiotropic activity of these new chemicals cannot be entirely excluded at the present time, the reason for this discrepancy could be multiple. In the present study, the mouse model was on a diet program for 12 week whereas in Febbraio’s studies, the CD36 null mice were fasted for 24 hour. Other differences may include gender and strain origin and differences in lipid metabolism. For instance, in the double CD36-ApoE knock-out mice, plasma TG were significantly different in male and female mice, depending on the diet [5]. In the present study we show that TG reduction was not affected by gender and genetic deletion. Alternatively, differences between a total disruption of the gene and a partial inhibition of the CD36 function with an IP administration of an inhibitor can be expected. For instance, CD36 expression in mice liver is low but the partial inhibitory activity of an administrated antagonist may be sufficient to reduce hepatic TG secretion [17]. The published observation that heterozygotes with partial CD36 deficiency have reduced plasma TG is in agreement with our findings and supports this possibility [40].

Increased plasma levels of TG and atherosclerosis are generally associated with impaired insulin action and glucose tolerance. Epidemiologic studies have implicated insulin resistance in both diabetes and coronary atherosclerosis [41]–[44]. Diabetic patients have areas of lipid rich plaques with greater macrophage infiltration and many of the processes that are implicated in plaque progression are amplified by the diabetic parameters. But, the molecular links between diabetes and atherosclerosis are still missing. Glycaemia alone stimulates macrophage accumulation but fails to promote macrophage proliferation at sites of lesions [45]. Defective insulin signaling is associated with the expression of CD36 and is enhanced via a CD36-dependent pathway [46], [47]. Increased CD36 expression has been shown to contribute to dyslipidemia and to be associated with insulin resistance and decreased glucose tolerance, suggesting that CD36 is involved in the physiopathology of insulin sensitivity. The present study supports this concept and shows that administration of small inhibitors of the CD36 functions improves insulin sensitivity and glucose tolerance in rodent animals. This activity was not animal model dependent and was not affected by genetic modifications. Therefore, anti-CD36 therapy may be beneficial to both atherogenic dyslipidemia and diabetes type2.

CD36 is expressed in both human and rat enterocytes and has been shown to be involved in the control of intestinal cholesterol and fatty acid uptake and secretion. CD36 is expressed in the small intestine and plays an important role in chylomicron metabolism and the production of large postprandial triglyceride rich particles [14,48, and 18]. The molecule is associated with the intestinal alkaline phosphatase in FA transport and the response to a fat diet [48] and specific defect in FA uptake in the proximal intestine of CD36^−/−^ mice is associated with reduced incorporation of FA in TG and a diminished TG secretion [14]. This concept was however challenged. Published observations have shown that CD36 genetic deletion does not affect intestinal lipid uptake and the efficient participation of CD36 in LCFA intestinal uptake was questioned [13], [18]. It was suggested that CD36 functions as a FA sensor and stimulates events that control FA metabolism rather than being directly involved in the lipid transit. In any case, our findings show that small inhibitors of the CD36 binding functions can significantly reduce the postprandial hypertriglyceridemia which follows a gastric olive challenge. Again, when compared to a complete deletion of the gene, which favor redundant mechanisms, a partial inhibition of CD36 functions may have different consequences. Our findings demonstrate that a selective down regulation of CD36 in the intestine reduces lipid intake and is beneficial to postprandial hypertriglyceridemia.

In conclusion, CD36 is generally recognized as an important lipid and FA receptor which plays a role in the metabolic syndrome and its associated cardiac events. The pleiotropic activity and the various molecular associations of this scavenger in different cells and tissues have however questioned its potential as a safe therapeutic target. Different published observations have indeed suggested that CD36 down regulation might not been beneficial due to redundant mechanisms or potential toxicity. The present study shows that it is possible to identify small molecules that can block the CD36 binding and uptake functions and that such antagonism can reduce atherosclerosis, postprandial hypertriglyceridemia and be beneficial for type II diabetes. Particularly, elevated postprandial hypertriglyceridemia is a metabolic parameter which is now recognized to be strongly associated with cardiovascular events and is independent of traditional cardiovascular risk factors [49]. Thus, CD36 might represent an attractive therapeutic target.
